# Using Focus Groups to Develop a Bone Health Curriculum for After-school Programs

**Published:** 2004-06-15

**Authors:** Christina D Economos, Sara C Folta, Jeanne P Goldberg, Lori P Marcotte

**Affiliations:** John Hancock Center on Physical Activity and Nutrition, Gerald J. and Dorothy R. Friedman School of Nutrition Science and Policy, Tufts University; Gerald J. and Dorothy R. Friedman School of Nutrition Science and Policy, Tufts University, Boston, Mass; Gerald J. and Dorothy R. Friedman School of Nutrition Science and Policy, Tufts University, Boston, Mass; Gerald J. and Dorothy R. Friedman School of Nutrition Science and Policy, Tufts University, Boston, Mass

## Abstract

**Introduction:**

Childhood behaviors influence peak bone mass and osteoporosis risk in later life. The after-school environment provides an opportunity to enrich a child’s learning and experience. Our objective was to gain a better understanding of the knowledge of, attitudes and beliefs about, and barriers to achieving bone health among children, parents, and after-school program leaders from low-income, ethnically diverse communities. Findings led to the development, implementation, and evaluation of a bone health curriculum in the after-school setting.

**Methods:**

Eight focus groups were conducted in three representative communities. Focus group participants included children aged six to eight years, parents of children aged six to eight, and after-school program staff. Transcripts and written notes from each session were reviewed and common themes were identified within each group.

**Results:**

Most adults had some understanding of osteoporosis, but did not recognize that childhood behaviors had a role in developing the disease. Program leaders raised concerns about their ability to implement a health program and recommended a flexible format. Parents and program leaders recognized the importance of maintaining a fun atmosphere.

**Conclusion:**

It is feasible to create a curriculum for a bone health program that meets the unique needs and interests of children and program leaders in the after-school setting. Addressing the needs, interests, and common barriers of the target population is an essential first step in curriculum development.

## Introduction

Osteoporosis is a childhood disease with adult consequences. Childhood behaviors, including diet and physical activity (1-4), have a major influence on the attainment of peak bone mass and the primary prevention of osteoporosis (5-11). The higher the peak bone mass in childhood, the more an individual can afford to lose in adulthood (12-14). The long-term benefits of increasing bone mineral density during childhood are compelling (15,16). A change in one negative standard deviation in bone mass may double fracture risk (17,18).

In the United States, there is a large gap between childhood behaviors known to help maximize bone health and what children actually do. National survey data estimate that more than half of girls aged six to 11 years are not meeting 100% of the 1989 Recommended Dietary Allowance for calcium, and nearly half of boys are not meeting this requirement (19). The gap between recommendations and intakes is difficult to reverse as children age (20,21). Of equal importance is that children of all ages do not obtain adequate levels of physical activity (22-25). Studies show that sedentary behavior increases and moderate physical activity decreases as children advance through elementary school (26,27) and that this decline continues into adolescence (27,28). Furthermore, girls are less likely to engage in physical activity than boys (27-29), and black children are less active than white children (28,29). The gap between the long-term effect of modifiable influences on bone health and the behaviors of millions of children suggests that cost-effective interventions to promote bone health in children are urgently needed.

After-school programs are ideal for complementing the school day with health education and physical activity. Several million children participate in after-school programs, and demand outstrips supply by a rate of approximately two to one (30). Furthermore, many programs lack adequate funding, and quality is highly variable (30). Curriculum-based interventions may enhance existing enrichment activities and provide structure to programs that are not highly developed. Reviews of nutrition and physical activity education curricula indicate that they can contribute to significant improvements in students’ knowledge, skills, and behavior, but that they must have certain characteristics to be effective (31-33). A health curriculum should be theory driven and should address children’s needs, interests, and concerns, in addition to their knowledge, attitudes, and beliefs (31-33). Addressing barriers to change is also important. This paper describes the design of a curriculum to promote bone health based on data obtained from focus group research to identify motivating factors, preferences, and barriers to change among children, parents, and after-school program leaders.

## Methods

Eight focus groups were conducted in three low- to moderate-income, multiethnic Massachusetts communities in the three months from November 1999 through January 2000. In total, 66 individuals participated. Participants included three groups of children aged six to eight years (N = 26; 70% white, 30% African American; 61% male); three groups composed of parents with children aged six to eight (N = 24; 80% white, 20% African American; 8% male); and two after-school program staff groups (N = 16; race and ethnicity not specified; 19% male). Of the 16 program staff who participated, two oversaw staff and program development and 14 taught. Focus groups took place at the after-school program sites and were led by two professional focus-group facilitators with expertise in conducting groups with children. Sessions typically lasted two hours and included six to 11 participants. Each adult participant received $30 and each child received a $20 gift certificate to a local toy store. Each session was recorded on audiotape for subsequent transcription; focus-group facilitators took additional notes.

Focus-group facilitators provided a brief introduction and invited parents and leaders to offer general opinions and comments about health education and strategies for engaging children in desired behaviors in after-school programs. Facilitators told children that the purpose of the meeting was to learn about what children like to eat and play. All groups were told there were no right or wrong answers. Facilitators explored knowledge, attitudes, beliefs, preferences, and barriers related to bone health and to the potential implementation of a curriculum that focused on bone health in the after-school environment.

The two facilitators systematically analyzed transcripts. Each one read the original transcripts to identify themes of each topic of discussion before collaborating on the summary report and submitting the report to an independent investigator. The investigator reviewed the transcripts and final report and recoded key phrases into a matrix constructed to conform to the project’s conceptual framework. Recoding key phrases into the matrix allowed for a more detailed understanding of the key themes identified by the facilitators and provided the ability to incorporate these themes into the project development.

The Institutional Review Board at Tufts University gave human subjects research approval for this project.

## Results

### Knowledge and awareness

As expected, the children had limited knowledge about bone health and the factors that affect it. Some understood the connection between bone health and drinking milk. Not surprisingly, they were generally aware of something called “calcium” but did not understand that it is a mineral or know where it is found in the diet: “It’s a kind of vitamin and cereal has it” was a typical response. After calcium was defined for them, many children commented, “Calcium makes you stronger, smarter, and helps you learn.”

As expected, none of the children understood “osteoporosis.” Among parents, knowledge of osteoporosis was mixed, whereas most after-school program leaders had a basic knowledge of what osteoporosis is and how to prevent it. In general, both parents and after-school program staff were aware of the effect of calcium and exercise on bone health and development.

### Attitudes, perceptions, and beliefs

Children showed little interest in understanding osteoporosis, but some interest in knowing how to make bones healthy and strong. Children appropriately associated bones with certain foods: “Bones make you think about dairy products.” Parents felt that nutrition played a critically important role in their child’s development. Among their chief concerns were getting their children to eat enough fruits and vegetables and limiting their intake of sweets and other “junk foods”: “I worry about the long-range effect of nutrition on them in their twenties, what will have been done by then.” “I like to make sure my kids get their vitamins every day…because I know they don’t eat right. They don’t eat enough vegetables.” “Other than genetics, nutrition is the number-one thing for your child’s health.”

Parents were less concerned that their children’s diets had enough calcium and did not consider osteoporosis a major health threat: “As long as they’re eating from the basic four food groups, I’m not worried.” “I always think of osteoporosis as an adult issue.” “I think I need the bone help more than them.”

Parents and after-school program leaders were both concerned about the amount of physical activity the children were getting. One parent commented, “He doesn’t get enough exercise — never. He’s healthy, but he has an interest in video games and anything electronic. I’m worried about down the road.” An after-school program leader observed, “If you talk to a gym teacher or watch a class, these kids aren’t in any shape at all. In my class, there are four or five kids who can’t run around the bases without stopping and huffing and puffing.”

### Preferences

Most children said they liked or drank milk. The perspectives of children and parents differed on the subject of physical activity. Children said that if given a choice, they would prefer physical activity, games, or sports during their free time: “I like to play tag and play games like when you pretend to be monsters and things…I’d rather play outside.” In contrast, parents consistently said that if left on their own, children would choose television and video games rather than physical activity. Parents demonstrated an awareness of the importance of physical activity and of their role in promoting it: “I do make them go outside, but it’s like kicking and screaming — they don’t like to go.”

### Barriers

After-school program leaders were concerned about the amount of planning required to implement a curriculum. Said one participant, “I’m a second-grade teacher. I have enough planning to do all day long. I don’t have the time.” Some after-school program leaders also expressed a desire for flexibility: “I think ideas would be better, because if you disagree with the format, then you’re going to come to some conflict with ‘Oh, I have to do this?’ Make it more optional. ‘You may want to do this, or you could do A, B, and C.’” “[It] just depends on the mood of the children what I’m going to do that day. If they’re fidgety, we go out and run around the park.”

While after-school program leaders recognized the need to provide guidance for children about healthy eating and exercise, they did not perceive health education as a priority: “I think health education is important but as [another participant] said, they get a lot of it during the day at school, and we’re more geared toward their social and emotional growth, socializing with other children and interacting with adults.”

Both parents and after-school program leaders expressed some concerns about the nutrition education component of the curriculum. They worried that the activity would replace the children’s already limited time for play and fun. One after-school program leader stated, “I don’t want it to be a bore for them. Especially since they’ve been in school all day long. I do think it’s important, but when they come to us, it’s time to let loose some steam.” A parent said, “I’m hoping they’ll come home and say ‘I had fun doing this and that today.’ If he says, ‘I *have* to go here,’ then he’s in the wrong place.”

Parents also expressed a concern that the nutrition education activity might be too academic: “It needs to be addressed for children as not so medical. It needs to be presented as fun.” “I think the calcium-focused activity would get old fast. You know: ‘Calcium again, I’m so sick of calcium.’”

Despite wanting their children to enjoy a break from academics, getting homework done during the after-school time was a high priority for parents. After-school program leaders felt pressure to make sure homework was complete by the time parents arrived to pick children up. One parent commented, “Homework has to be the first priority. I get home too late to get it done with him.” An after-school leader said, “I know my parents: they want [their children] to get their homework done.”

After-school program leaders consistently and poignantly expressed their concern that they might not know enough to effectively teach the bone-health curriculum. They were afraid they would be embarrassed if they could not answer a child’s question. “I’m not saying I’m ignorant about osteoporosis, but I’m not as knowledgeable as I’d like to be.” “[I would want] more knowledge about osteoporosis, questions the kids would ask us, so we could have answers for them.” Parents also expressed concern about the ability of the after-school program leaders to implement the curriculum: “The after-school teachers would need training. They’re capable, but need training.” While an extensive training program was proposed, most after-school program leaders suggested that only minimal training would be possible because of limited available time. Because of high staff turnover rates in the after-school setting, they also voiced an interest in ongoing oversight and support so the curriculum could continue even if trained leaders left the after-school program.

### Shaping the curriculum

Curriculum development relied heavily on information obtained in the focus groups. To respond to the needs of program leaders in the after-school setting, short and simple lessons were designed with alternate activity options, tips for implementation, and ideas for modifying games. Curriculum components could complement regular program activities without interfering with priorities such as homework. Ongoing support was offered via newsletters, and research staff were available to assist new leaders during the year.

From the outset of the project, the objective was to package both a physical-activity component and a nutrition education component so that the children would have fun while learning. The children know the project as “The Bones Club.” To address the desire expressed in focus groups to allow children to use after-school time for fun activities that would enable them to socialize and “let off some steam” and to fulfill the objective of offering simple, non-academic language, the physical activity component was named “Let’s Play.” Activities identified as favorites with the children were adapted to weight-bearing activities (similarly titled “Let’s Run” and “Let’s Jump”). Because after-school program leaders indicated that they operate in a wide variety of physical environments, all games included simple modifications to accommodate play in different environments.

Likewise, the nutrition education component was named “Let’s Explore” to reflect some of the preferred activities of children and to emphasize both teamwork and fun. During the focus groups, children indicated an interest in reading, and after-school program leaders reported “circle time” as a common component of the after-school day. Age-appropriate books were provided to support the learning themes of the “Let’s Explore” lessons. Many after-school program leaders expressed concern that they may not know enough about bone health to teach the curriculum effectively. To begin to address this, an appendix was included with each section of the written curriculum that answered commonly asked questions and provided a quick reference guide for additional resources.

Evidence shows that nutrition education programs and curricula targeted at elementary-aged children are more effective when they include a family component (33). Some parents received newsletters that corresponded to curriculum units to reinforce after-school program lessons at home. Newsletters included quick and easy recipes and physical activity tips that took into account the time constraint that was mentioned as a barrier in the focus groups. Parents also were given a directory that allowed them to leverage their own limited resources by using nutrition, physical activity, and health resources available in their communities.

## Discussion

This study demonstrates how focus groups can be used to shape a curriculum to meet the needs of after-school program leaders, parents, and children so that maximum buy-in and learning can occur. Of particular importance, focus groups can identify key barriers to implementing the curriculum that might otherwise go unnoticed. Perhaps the most important barrier was that health education was not considered a priority by either parents or after-school program leaders. To succeed, the curriculum would need to focus on fun for the children and ease of implementation for the program leaders. The curriculum was designed to be short and flexible so it would not replace activities that were considered a priority.

Parents and program leaders indicated limited confidence in promoting health, particularly nutrition, to children. Still, program leaders believed they could incorporate such a program into their existing after-school program structure and implement it as long as they are given adequate support.

Not surprisingly, children were not interested in osteoporosis the disease, but they did want to learn about how bones move and what they could do to grow big and strong. This perception confirmed that it is possible to engage even very young children in a health topic if the topic is presented at their level of comprehension and if it appeals to their interests.

The children who participated in the focus groups were young. Sometimes they were wonderfully direct and open, and at other times their responses were colored by the need for peer acceptance. In this series of focus groups, their responses about likes and dislikes differed from those of their parents. For instance, children overwhelmingly expressed a preference for active games or sports over video games, but parents reported difficulty in engaging children in outdoor play. This observation confirms the need to conduct focus groups that include both children and parents to obtain a more balanced picture of preferences and behaviors.

The inconsistency between children’s reported desire for physical activity and parents’ reports that children engage in sedentary behaviors if given a choice is difficult to reconcile. Possibly, while children may like the idea of physical activity, they are reluctant to engage in it once they have started other activities. Several factors may draw children to activities that are more sedentary. In the focus groups, parents noted the ubiquity of televisions and computer games in their homes. In addition, cold weather and early darkness were also mentioned as serious barriers to outdoor play. Regardless of these perceived barriers, children participated willingly when provided with the types of physical activities in the after-school programs that both the children and the program leaders agreed were fun.

Focus groups do not provide data that are generalizable to other populations, but they can be a time-efficient and cost-effective method for identifying attitudes, beliefs, and barriers toward health behaviors among defined target populations. Through an interactive discussion led by trained professionals, it is possible to identify information that is critical to program success and that might not be uncovered in survey research. For example, the permissive environment allowed after-school leaders to openly describe their perceptions of their limited knowledge about osteoporosis and bone health, which, if not addressed, could limit their ability to implement the curriculum and could consequently hinder the success of the program.

Response to the bone-health curriculum has been enthusiastic. More than 50 after-school programs in Massachusetts and Rhode Island have implemented it successfully, and it has been well-accepted by after-school program leaders, parents, and children. After-school program leaders report that the curriculum has enhanced their programs and has had the unexpected benefit of improving their relationships with the children. They indicate that children enjoy being in the “Bones Club” and having something to call their own. Participation is optional, but remains at a high level, and dropout rates related to dissatisfaction are extremely low (less than 1%). Dropout is linked almost exclusively to children leaving the after-school program or the school district itself.

An environment that fosters the development of behaviors to promote bone health can contribute to positive habits that children will adopt before entering their preteen years, when peer influences gain power. After-school programs have been an underused setting for health interventions. As they grow in number, they provide an opportunity to use time that traditionally has been difficult to fill consistently with appropriate physical and cognitive activities for all children who attend them. Health interventions that include an academic and a physical-activity component are difficult to implement given the varied experience of leaders and the lack of funds for training and technical support. Limited staff, high turnover rates, and competing demands on program time are major barriers. Curricula based on formative research can overcome these barriers, help to improve the health of children, and prevent chronic disease later in life.
